# Insights into CO_2_ Fixation Pathway of *Clostridium autoethanogenum* by Targeted Mutagenesis

**DOI:** 10.1128/mBio.00427-16

**Published:** 2016-05-24

**Authors:** Fungmin Liew, Anne M. Henstra, Klaus Winzer, Michael Köpke, Sean D. Simpson, Nigel P. Minton

**Affiliations:** a BBSRC/EPSRC Synthetic Biology Research Centre (SBRC), School of Life Sciences, University Park, The University of Nottingham, Nottingham, United Kingdom; b LanzaTech Inc., Skokie, Illinois, USA

## Abstract

The future sustainable production of chemicals and fuels from nonpetrochemical resources and reduction of greenhouse gas emissions are two of the greatest societal challenges. Gas fermentation, which utilizes the ability of acetogenic bacteria such as *Clostridium autoethanogenum* to grow and convert CO_2_ and CO into low-carbon fuels and chemicals, could potentially provide solutions to both. Acetogens fix these single-carbon gases via the Wood-Ljungdahl pathway. Two enzyme activities are predicted to be essential to the pathway: carbon monoxide dehydrogenase (CODH), which catalyzes the reversible oxidation of CO to CO_2_, and acetyl coenzyme A (acetyl-CoA) synthase (ACS), which combines with CODH to form a CODH/ACS complex for acetyl-CoA fixation. Despite their pivotal role in carbon fixation, their functions have not been confirmed *in vivo*. By genetically manipulating all three CODH isogenes (*acsA*, *cooS1*, and *cooS2*) of *C. autoethanogenum*, we highlighted the functional redundancies of CODH by demonstrating that *cooS1* and *cooS2* are dispensable for autotrophy. Unexpectedly, the *cooS1* inactivation strain showed a significantly reduced lag phase and a higher growth rate than the wild type on H_2_ and CO_2_. During heterotrophic growth on fructose, the *acsA* inactivation strain exhibited 61% reduced biomass and the abolishment of acetate production (a hallmark of acetogens), in favor of ethanol, lactate, and 2,3-butanediol production. A translational readthrough event was discovered in the uniquely truncated (compared to those of other acetogens) *C. autoethanogenum acsA* gene. Insights gained from studying the function of CODH enhance the overall understanding of autotrophy and can be used for optimization of biotechnological production of ethanol and other commodities via gas fermentation.

## IMPORTANCE

Gas fermentation is an emerging technology that converts the greenhouse gases CO_2_ and CO in industrial waste gases and gasified biomass into fuels and chemical commodities. Acetogenic bacteria such as *Clostridium autoethanogenum* are central to this bioprocess, but the molecular and genetic characterization of this microorganism is currently lacking. By targeting all three of the isogenes encoding carbon monoxide dehydrogenase (CODH) in *C. autoethanogenum*, we identified the most important CODH isogene for carbon fixation and demonstrated that genetic inactivation of CODH could improve autotrophic growth. This study shows that disabling of the Wood-Ljungdahl pathway via the inactivation of *acsA* (encodes CODH) significantly impairs heterotrophic growth and alters the product profile by abolishing acetate production. Moreover, we discovered a previously undescribed mechanism for controlling the production of this enzyme. This study provides valuable insights into the acetogenic pathway and can be used for the development of more efficient and productive strains for gas fermentation.

## INTRODUCTION

Acetogenic bacteria employ the Wood-Ljungdahl pathway (WLP) to fix CO_2_ (in the presence of H_2_) and CO into the central metabolite acetyl coenzyme A (acetyl-CoA). It is the only linear CO_2_ fixation pathway known and the most thermodynamically efficient pathway in acetate synthesis (1). As a consequence, the WLP is a prime candidate for the earliest autotrophic pathway in the origin of life (2). Terrestrial production of acetate by acetogens is estimated to be at least 10^13^ kg/annum, accounting for more than 20% of the fixed carbon on Earth, highlighting their significant role in the global carbon cycle (3). The ability to fix C_1_ gases also makes acetogens attractive process organisms for the production of chemicals and fuels. Fermentation processes that recycle waste gases from industrial processes or syngas generated from any biomass source are on the verge of commercialization (4) and offer significant greenhouse gas emission savings (5) to meet the climate goals under the Paris Agreement (6).

Crucial to the function of the WLP are the enzymes carbon monoxide dehydrogenase (CODH) and acetyl-CoA synthase (ACS). CODH catalyzes the interconversion of CO and CO_2_ according to the equation CO + H_2_O ←→ CO_2_ + 2H^+^ + 2e^−^. CO is a potent electron donor (CO_2_/CO reduction potential of −558 mV [pH 7.0]) (7). Relatively few acetogens are, however, able to grow on CO alone because of growth inhibition resulting from the sensitivity of metal-containing enzymes to CO (8). CODH can also form a bifunctional complex with the ACS that couples the reduction of CO_2_ and the formation of acetyl-CoA. This unique enzyme has been extensively studied at the protein level (9–11), but *in vivo* and genetic studies of CODH in acetogens are lacking.

*Clostridium autoethanogenum*, a model acetogen, is able to grow on CO as a sole carbon and energy source and produce ethanol, acetate, 2,3-butanediol, and lactate (12, 13). Whole-genome sequencing of this acetogen revealed the presence of three putative CODHs: CAETHG_1620-1621 (*acsA*), CAETHG_3005 (*cooS1*), and CAETHG_3899 (*cooS2*) (14). *acsA* is in an 18-kbp cluster with genes of the WLP and is predicted to encode the CODH component of the CODH/ACS complex, while *cooS1* is localized upstream of a gene encoding a putative 4Fe-4S ferredoxin binding domain-containing protein and ferredoxin oxidoreductase. *cooS2* appears to be an orphan. Transcriptomic studies have shown that all three genes are expressed, with *acsA* and *cooS1* being among the most highly expressed genes within the genome (15, 16).

Given the number of CODHs within *C. autoethanogenum*, it is unknown whether all of them are essential for autotrophic growth and are true isozymes or have distinct functions. Here, we addressed this question by independently inactivating all three CODH-encoding genes by ClosTron mutagenesis (17–19) and then examined the impact on autotrophy and product formation. The mutant strain in which *acsA* was inactivated is particularly interesting because it essentially has a disabled WLP and, as a consequence, displays a radically different metabolite distribution, including the complete abolition of acetate formation.

Intriguingly, compared to the *acsA* genes of other acetogens, that of *C. autoethanogenum* uniquely contains an in-frame TGA stop codon. The encoded enzyme is therefore predicted to be truncated by some 231 amino acids and the gene effectively split in two: CAETHG_1621 and CAETHG_1620. By using FLAG-tagged *acsA* variants, the apparent *acsA* truncation event was investigated.

## RESULTS

### *acsA* is essential for autotrophy in *C. autoethanogenum.*

**FIG 1:**
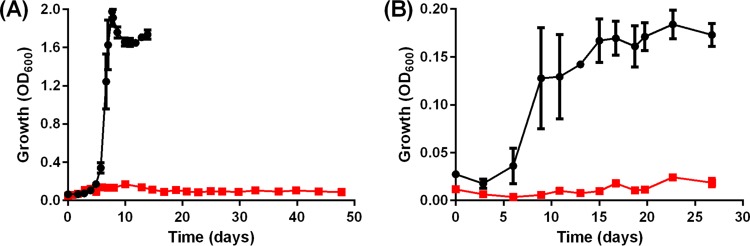
The *acsA* KO strain is unable to grow autotrophically on 200 kPa CO (A) or 130 kPa H_2_ plus 70 kPa CO_2_ (B). Symbols: black circles, WT (*n =* 4 for CO; *n =* 3 for H_2_-CO_2_); red squares, *acsA* KO strain (*n =* 3). Error bars show the standard error of the mean.

To determine the roles of *acsA* in supporting autotrophy in *C. autoethanogenum*, we first inactivated the gene by ClosTron mutagenesis (17–19), which resulted in an *acsA* knockout (KO) strain (see Fig. S1 in the supplemental material). Both the KO and wild-type (WT) strains were subjected to autotrophic batch growth on either CO or H_2_-CO_2_ (2:1) to assess the role of *acsA* in catalyzing CO oxidation and/or fixation of CO_2_ (using H_2_ as a reductant). As shown in Fig. 1A, the *acsA* KO strain displayed no sign of growth on CO following 48 days of incubation, whereas the WT reached an optical density at 600 nm (OD_600_) of 1.98 after day 8. Under H_2_-CO_2_ conditions, the *acsA* KO strain was unable to grow within 27 days, whereas the WT achieved stationary phase (OD_600_ of 0.17) after ~day 15 (Fig. 1B). In an attempt to restore autotrophy, plasmid pMTL83151-P_acsA_-acsA^full^ containing *acsA* was conjugated into the *acsA* KO strain to generate a complementation strain. The complementation strain was able to restore growth and acetate formation on CO, albeit growing to an OD_600_ of 1.10 after a growth lag phase of ~21 days (see Fig. S2 in the supplemental material) and generating 15% less acetate (*P* = 0.009). The growth characteristics of the plasmid control strain (harboring pMTL83151-P_acsA_) were generally similar to those of the WT, except that the lag phase was longer by 5 days (Fig. 1A; see also Fig. S2 in the supplemental material).

### Inactivation of *acsA* abolishes acetate formation during heterotrophic growth.

**FIG 2:**
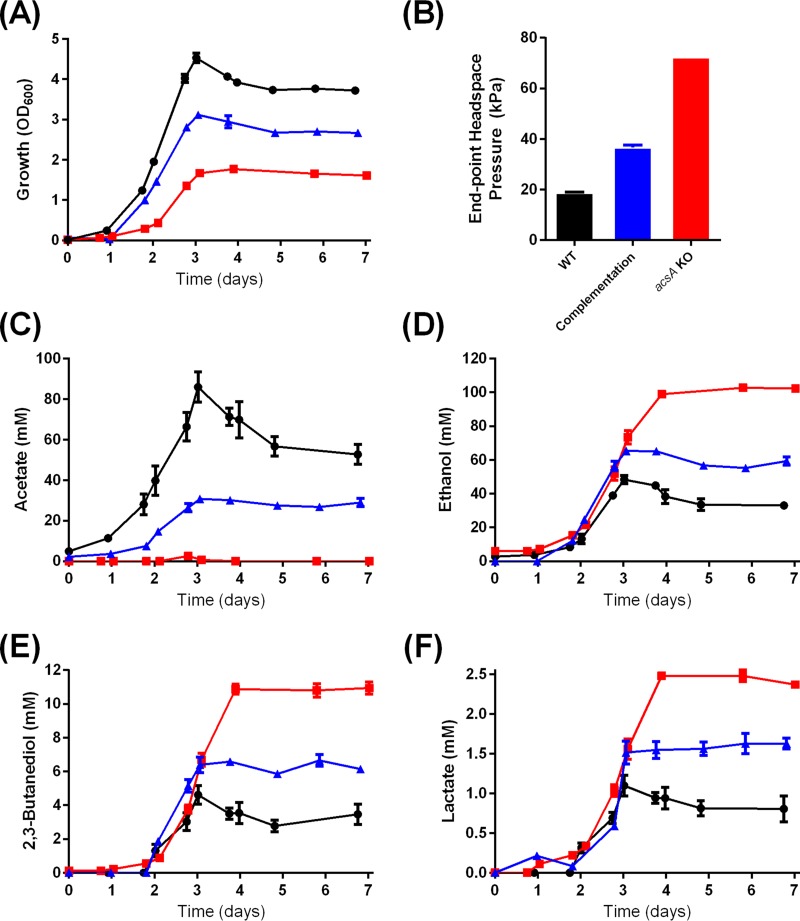
Growth, headspace pressure, and metabolite profiles of the *C. autoethanogenum* WT, *acsA* KO, and complementation strains on 10 g/liter fructose. Panels: A, growth profile; B, headspace pressure profile; C, acetate profile; D, ethanol profile; E, 2,3-butanediol profile; F, lactate profile. Black, WT; red, *acsA* KO strain; blue, complementation strain. *n =* 3. Error bars show the standard error of the mean.

In order to gain an insight into the role of *acsA* during heterotrophic growth, we next investigated the growth and product profile of the *acsA* KO strain on fructose. The *acsA* KO strain fully exhausted the supplemented 10 g/liter fructose but reached a 61% lower OD_600_ (*P* < 0.0001) and exhibited a longer growth lag phase than the WT (Fig. 2A). The 3.8-fold higher headspace pressure recorded at the end of the growth experiment with the *acsA* KO strain (Fig. 2B) indicates that more CO_2_ was being released from fructose metabolism as a result of the organism’s inability to reassimilate the released CO_2_ as a consequence of its dysfunctional WLP.

In terms of metabolite production, the *acsA* KO strain only transiently produced a trace amount of acetate (2.6 mM on day 2.8) while growing on fructose (Fig. 2C). In contrast, the WT strain generated 86.0 mM acetate under the same conditions (Fig. 2C). In the *acsA* KO strain, most of the carbon from fructose was diverted from acetate toward reduced products ethanol and 2,3-butanediol and toward lactate, as evident in increases of 113, 138, and 125%, respectively, relative to the WT (Fig. 2D to F). Similar to autotrophic growth on CO (mentioned earlier), the plasmid expression of *acsA*^full^ in the *acsA* KO strain partially restored the phenotypes of heterotrophic growth by reducing the growth lag phase to WT levels and increased the OD_600_ from 1.77 (*acsA* KO) to 3.11, which is 69% of the WT level (Fig. 2A). The complementation strain also synthesized 30.78 mM acetate (up from the 2.5 mM of the *acsA* KO), 65.35 mM ethanol (down from the 102.74 mM of the *acsA* KO), and 6.66 mM 2,3-butanediol (down from 10.95 mM) (Fig. 2C to E). Plasmid expression of *acsA* (pMTL83151-P_acsA_-acsA^full^) in the WT had only minimal effects on growth and metabolite production during growth on CO (see Fig. S3 in the supplemental material) or under heterotrophic growth conditions (see Fig. S4 in the supplemental material).

### Translational readthrough of *acsA.*

Genome sequencing of *C. autoethanogenum* (14, 20, 21) revealed the presence of an internal TGA stop codon within the *acsA* gene, splitting the gene into coding sequences (CDSs) CAETHG_1621 (1,203 bp) and CAETHG_1620 (684 bp) (see Fig. S5 in the supplemental material). Sanger sequencing confirmed the presence of the TGA stop codon (data not shown).

**FIG 3:**
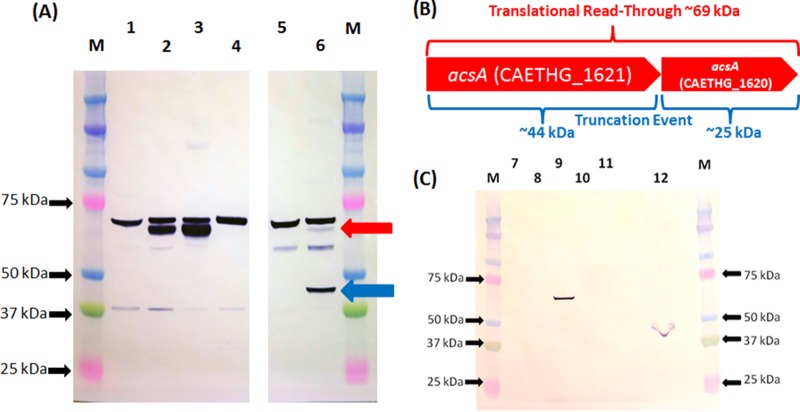
Examination of AcsA translation pattern with FLAG-tagged protein in *C. autoethanogenum* and *E. coli*. (A) Western blot analysis of *C. autoethanogenum* transconjugant crude lysates. Lanes: M, Bio-Rad kaleidoscope Precision Plus protein ladder; 1, 13 µg of soluble lysate of pMTL8315-P_acsA_ plasmid control; 2, 13 µg of soluble lysate of pMTL83151-P_acsA_-acsA(TGA)-FLAG; 3, 13 µg of soluble lysate of pMTL83151-P_acsA_-acsA(TCA)-FLAG; 4, 13 µg of soluble lysate of pMTL83151-P_acsA_-acsA(TAA)-FLAG; 5, 6.7 µg of insoluble lysate of pMTL83151-P_acsA_ plasmid control; 6, 6.7 µg of insoluble lysate of pMTL83151-P_acsA_-FLAG-acsA(TGA). The red arrow indicates the position of a mature 69-kDa protein; the blue arrow indicates the position of the larger 44-kDa truncated protein. The ca. 70-kDa band present in all crude lysates from *C. autoethanogenum* is a consequence of nonspecific binding of the anti-FLAG antibody to a native *C. autoethanogenum* protein, most likely DnaK (encoded by CAETHG_2891), which shares 7/8 amino acid identity with FLAG and is of the appropriate predicted size. (B) Schematic showing the expected protein sizes of *C. autoethanogenum* AcsA in the event of translational readthrough or truncation. (C) Western blot analysis of *E. coli* transformant crude lysates. Lanes: M, Bio-Rad kaleidoscope Precision Plus protein ladder; 7, 15 µg of soluble lysate of pMTL83151-P_acsA_ plasmid control; 8, 15 µg of soluble lysate of pMTL83151-P_acsA_-acsA(TGA)-FLAG; 9, 15 µg of soluble lysate of pMTL83151-P_acsA_-acsA(TCA)-FLAG; 10, 15 µg of soluble lysate of pMTL83151-P_acsA_-acsA(TAA)-FLAG; 11, 39 µg of soluble lysate of pMTL83151-P_acsA_-FLAG-acsA(TGA); 12, 38 µg of insoluble lysate of pMTL83151-P_acsA_-FLAG-acsA(TGA).

By fusing a FLAG tag to the N or C terminus of AcsA, the *acsA* translation pattern in *C. autoethanogenum* and *Escherichia coli* was investigated by Western blot analysis. In addition, the TGA stop codon was replaced with a TCA or TAA codon by splicing by overhang extension PCR (SOE-PCR). Modified genes were expressed from plasmids. The truncation of the 69-kDa full-length AcsA protein can result in proteins of 44 and/or 25 kDa (Fig. 3B). With a C-terminal FLAG tag, a 69-kDa protein was detected in *C. autoethanogenum* crude lysates (Fig. 3A), while a 25-kDa protein band was absent. With the N-terminal FLAG tag variant, the 44- and 69-kDa proteins were both detected, with the intensity of the 44-kDa protein band being higher. When the TGA codon was replaced with a TCA codon, only the 69-kDa protein was detected. These results indicate that translational readthrough of the *acsA* TGA codon occurs with low frequency in *C. autoethanogenum* and there is no independent translation of the downstream CDS.

*E. coli* is a model microorganism for the study of selenocysteine synthesis and is capable of selenocysteine incorporation (22, 23). Lysates of *E. coli* cells that expressed *acsA* variants encoding either N- or C-terminally FLAG-tagged proteins failed to produce the 69-kDa full-size AcsA product (Fig. 3C). Instead, the 44-kDa truncated protein was detected. However, the *acsA* variant with a TCA serine codon successfully generated the full-size ~69-kDa protein (Fig. 3C). Replacement of the internal stop codon with a TAA stop codon completely eliminated the translation of the full-size 69-kDa AcsA peptide in both *C. autoethanogenum* and *E. coli* (Fig. 3A and C).

### *cooS1* and *cooS2* are dispensable for autotrophy.

**FIG 4:**
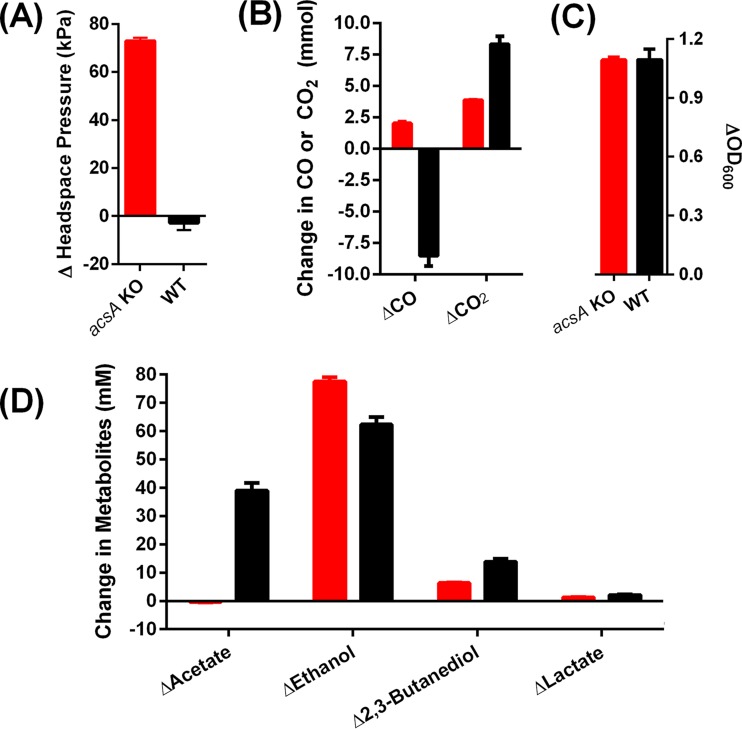
Changes in biomass, headspace, and metabolite levels between the start and finish of a mixotrophic-growth experiment with the *acsA* KO and WT strains on 10 g/liter fructose and 200 kPa CO. Panels: A, change in headspace pressure; B, change in headspace CO or CO_2_; C, change in growth based on OD_600_; D, change in metabolites. Columns: red, *acsA* KO strain; black, WT. *n =* 3. Error bars show the standard error of the mean.

Besides *acsA*, the genome of *C. autoethanogenum* contains two additional putative CODHs: *cooS1* and *cooS2*. These are unable to compensate for *acsA* and are therefore predicted to be monofunctional, as shown above. Accordingly, the *acsA* KO strain would be expected to grow on fructose (10 g/liter) and oxidize CO (22.4 mmol) into CO_2_ by using the unperturbed *cooS1*- and/or *cooS2*-encoded CODHs. In experiments that compared the *acsA* KO strain to the WT grown mixotrophically on CO plus fructose, the *acsA* KO strain generated 2.03 mmol of CO, while the WT control consumed 8.52 mmol of CO (Fig. 4B). Both strains completely exhausted the fructose at the end of the experiment (data not shown)

**FIG 5:**
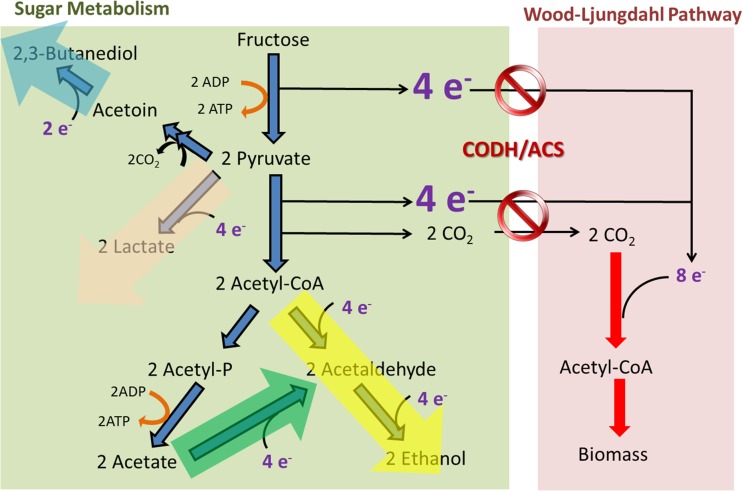
Inactivation of CODH/ACS in *C. autoethanogenum* generates excess reducing equivalents that are consumed in biochemical reactions that lead to ethanol, 2,3-butanediol, and lactate formation.

In the absence of a WLP, the consumption of 10 g/liter fructose (or an absolute amount of 2.8 mmol) is expected to produce a maximum CO_2_ level of 5.6 mmol (1 mol of fructose yields 2 mol of pyruvate, which in turn is decarboxylated into acetyl-CoA with the concomitant release of 2 mol of CO_2_; Fig. 5). In acetogens, a large proportion of the released CO_2_ is fixed into acetyl-CoA. Since the WT produced 8.3 mmol of CO_2_ during mixotrophic growth on 2.8 mmol fructose and 8.52 mmol of CO (Fig. 4B), which is greater than the theoretical maximum of 5.6 mmol of CO_2_, CO oxidation must have occurred, as opposed to the direct use of CO in the carbonyl branch of the WLP. These results indicate that while the WT strain is able to oxidize CO during mixotrophic growth, neither *cooS1* nor *cooS2* in the *acsA* KO strain is able to catalyze CO oxidation. Instead, the physiological roles of these monofunctional CODHs under mixotrophic growth conditions may lie in the direction of CO_2_ reduction since additional CO was produced by the *acsA* KO strain.

**FIG 6:**
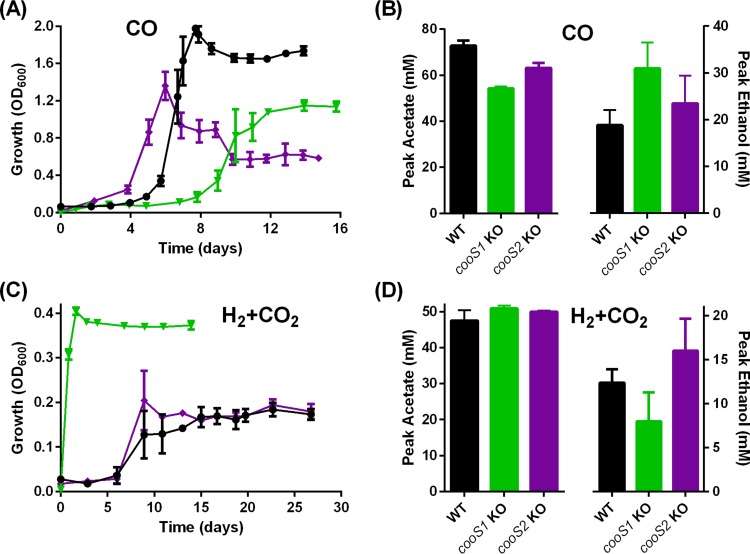
Growth and metabolite profiles of the *C. autoethanogenum* WT and *cooS1* and *cooS2* KO strains on CO or H_2_-CO_2_. Panels: A, growth profile on CO; B, metabolite profile on CO; C, growth profile on H_2_-CO_2_; D, metabolite profile on H_2_-CO_2_. Columns: black, WT (*n =* 4 for CO, *n =* 3 for H_2_-CO_2_); green, *cooS1* KO strain (*n =* 3); purple, *cooS2* KO strain (*n =* 3). Error bars show the standard error of the mean.

By ClosTron mutagenesis, *cooS1* and *cooS2* of *C. autoethanogenum* were independently inactivated to assess their roles in autotrophy (see Fig. S1 in the supplemental material). When grown on pure CO, the *cooS1* KO strain displayed some growth deficiencies, including an ~2.9-day-longer lag phase, and achieved a 42% lower OD_600_ (*P* = 0.0002) than the WT (Fig. 6A). It produced 25% less acetate (*P* = 0.0004) and a similar amount of 2,3-butanediol (data not shown) but 64% more ethanol (not statistically significant) than the WT (Fig. 6B). The reduced growth of the *cooS1* KO strain in the presence of CO may result in the accumulation of excess reducing equivalents that are consumed in ethanol biosynthetic pathways. Remarkably, under H_2_-CO_2_ conditions, the *cooS1* KO strain is able to grow without an apparent lag phase and also reaches twice the OD_600_ of the WT (Fig. 6C).

Unlike the autotrophic growth of the *cooS1* KO strain with CO, that of the *cooS2* KO strain is not significantly affected. The *cooS2* KO strain even showed a mild decrease in the growth lag phase but a lower final OD_600_ in the presence of CO than the WT (Fig. 6A). This result, combined with a lack of impact on H_2_-CO_2_ autotrophic growth (Fig. 6C), suggests that *cooS2* is not heavily involved in autotrophy of *C. autoethanogenum*.

## DISCUSSION

Understanding the fundamentals of C_1_ metabolism in acetogens is a prerequisite for their further development as a chassis for the sustainable production of chemicals and fuels from waste gases. By independently disrupting all three CODH isogenes in *C. autoethanogenum*, we investigated their roles in supporting autotrophy. The complete absence of growth of the *acsA* KO strain on CO or H_2_-CO_2_ demonstrated that *acsA* is absolutely essential for autotrophy under both CO and H_2_-CO_2_ conditions and that unperturbed *cooS1* and *cooS2* are unable to compensate for the loss of *acsA* function. In *Methanosarcina acetivorans*, which has two CODH/ACS paralogs, deletion studies showed that the microorganism can grow autotrophically in CO when one of these genes was deleted but not when both were deleted (24).

During glycolysis, 1 mol of hexose sugar is metabolized to 2 mol of acetyl-CoA, 2 mol of CO_2_ (generated during the pyruvate:ferredoxin oxidoreductase reaction), and 8 reducing equivalents (Fig. 5). Acetogens such as *C. autoethanogenum* utilize the 8 reducing equivalents to reassimilate the released 2 mol of CO_2_ into an additional acetyl-CoA, resulting in complete carbon conversion (3). Based on this, a concept called acetogenic mixotrophy was recently proposed to improve biofuel and biochemical yields (25). The *acsA* KO strain constructed in this study provides a unique opportunity to examine the effect of a disabled WLP on a microbe that normally performs acetogenic mixotrophy. The growth of the *acsA* KO strain on fructose was significantly impaired, highlighting the role of CODH/ACS in biomass formation during heterotrophic growth.

Acetate production, which generates an ATP per molecule of acetyl-CoA via substrate level phosphorylation, is one of the hallmarks of acetogens (3). Hence, it is surprising that the *acsA* KO strain with unperturbed phosphotransacetylase (*pta*) and acetate kinase (*ack*) genes only transiently produced trace amount of acetate while growing on fructose. Acetate is often viewed as an undesirable by-product during biofuel or commodity chemical production, so there are reports in the literature of attempts to engineer strains that produce no acetate. Most studies tried to block carbon flow by inactivating the key acetate-forming enzyme-encoding genes *pta* and/or *ack* (e.g., in *Clostridium acetobutylicum* [26], *Clostridium tyrobutyricum* [27], or *E. coli* [28]), but acetate production was not abolished and growth was reduced instead.

Instead of producing acetate and biomass, the *acsA* KO strain produced significantly more ethanol, 2,3-butanediol, and lactate than the WT while growing on fructose. Production of these products serves as an alternative sink to the disabled WLP (due to *acsA* inactivation) for reducing equivalents generated by glycolysis (Fig. 5), resulting in a redistribution of electron and carbon fluxes. By a similar approach, acetate production could be significantly reduced and carbon redistributed to ethanol when inactivating hydrogenase maturation proteins (and, therefore, hydrogenase activity) in *Clostridium thermocellum* (29). Certain acetogens, including *C. autoethanogenum*, harbor the enzyme aldehyde:ferredoxin oxidoreductase (14), which could reduce acetic acids into acetaldehyde using reduced ferredoxin, followed by the formation of ethanol via ethanol dehydrogenase. Accordingly, the lack of acetate production in the *acsA* KO strain could be due to an increase in the rate of acetic acid reduction. Collectively, our results demonstrated that although a functional WLP allows higher carbon utilization efficiency during heterotrophic growth by fixing CO_2_ into biomass and acetate, the biosynthesis of ethanol, 2,3-butanediol, and lactate is significantly reduced in the absence of an additional reductant (e.g., H_2_ or CO).

The *acsA* gene of *C. autoethanogenum* is uniquely truncated because of an internal TGA stop codon. The *acsA* genes of five other clostridial acetogens, *Clostridium ljungdahlii* (30), “*Clostridium ragsdalei*” (12), *Clostridium carboxidivorans* P7 (31), *Clostridium aceticum* (32), and *Clostridium difficile* 630 (33), with identical operon topologies showed no gene-splitting event (see Fig. S5A in the supplemental material). Instead, a TCA serine codon is present in these acetogens. Given the essential role of *acsA* in acetogenesis, it was of interest to determine whether posttranscriptional mechanisms allowed translational readthrough beyond the internal stop codon in *C. autoethanogenum*. One such mechanism is the incorporation of selenocysteine at the TGA codon, which is generally reliant on a characteristic bacterial selenocysteine insertion sequence (bSECIS) immediately downstream of the stop codon. A bSECIS element was not detected in the *C. autoethanogenum* acsA gene by using an algorithm (34), but such an element was uncovered via manual examination (see Fig. S5B in the supplemental material). The catalytic activities of many selenoproteins are often superior to those of their cysteine-dependent counterparts (35).

By investigating the translational pattern of FLAG-tagged AcsA protein by Western blot analysis, we showed that a partial and inefficient translational readthrough event occurs in *C. autoethanogenum* as formation of the truncated AcsA protein is the main product. It is not clear whether the ability of *C. autoethanogenum* to generate mature and truncated AcsA is a novel regulatory mechanism for an as-yet-unknown physiological purpose or whether it poses a handicap that hinders acetogenesis. A search of the curated genome (21) revealed 52 CDSs with TGA stop codons closely followed by an in-frame CDS (see Table S1 in supplemental material), including a selenocysteine formate dehydrogenase (36). Thus, *acsA* may not be the only *C. autoethanogenum* gene with an internally translated stop codon.

In contrast to *C. autoethanogenum*, plasmid expression of the FLAG-tagged AcsA protein in *E. coli* (a model organism for selenocysteine formation and incorporation) did not result in the formation of a translational readthrough product. This result indicates that the translational readthrough event in *acsA* is *C. autoethanogenum* specific and is unlikely to involve selenocysteine incorporation. Stop codon readthrough is not uncommon and especially prevalent for the UGA codon, while UAA is a more efficient translational stop signal (37). When the TAA stop codon of *acsA* replaced the internal TGA stop codon, translation of the full-length AcsA peptide in *C. autoethanogenum* and *E. coli* was completely eliminated. Stop codon readthrough depends on the competition between a release factor and a near-cognate tRNA (37). The genome of *C. autoethanogenum* encodes one tRNA with anticodon 5ʹ-CCA-3ʹ (CAETHG_R0046) for tryptophan.

In this study, inactivation of either *cooS1* or *cooS2* moderately lowered biomass formation during growth on CO but did not negatively impact growth on H_2_-CO_2_, highlighting functional redundancies in acetogens that harbor multiple CODH isogenes. The orthologous *cooS1* and *cooS2* genes of *C. ljungdahlii* were reported to be expressed at lower levels when the bacterium was grown autotrophically on CO rather than heterotrophically on fructose, leading the authors to hypothesize that monofunctional CODHs do not significantly contribute to the oxidation of CO, but it is the bifunctional CODH/ACS complex that is mainly responsible (16, 38). In agreement with this hypothesis, during mixotrophic growth on fructose plus CO, *C. autoethanogenum* WT, but not the *acsA* KO strain (which still carries the functional *cooS1* and *cooS2* genes), was able to oxidize CO.

During growth on H_2_-CO_2_, the *cooS1* KO strain, surprisingly, grew without an apparent lag phase and reached twice the OD_600_ of the WT strain. Since the reduced [CO] moiety is enclosed within the CODH/ACS complex to lower internal thermodynamic barriers during acetyl-CoA synthesis (39–41), the presence of another CODH may act as a competitor for CO_2_. The inactivation of *cooS1* would, therefore, increase the efficiency of the CODH/ACS enzyme complex, leading to the observed enhanced growth on H_2_-CO_2_, and may represent a metabolic engineering strategy to improve gas utilization efficiency in acetogens that harbor multiple CODH-encoding isogenes.

## MATERIALS AND METHODS

### Bacterial strains and growth conditions.

For the bacterial strains used in this study, see Table S2 in the supplemental material. The *E. coli* strains used for general plasmid propagation, cloning, and conjugation were cultivated at 37°C in LB medium in the presence of antibiotics (25 µg/ml chloramphenicol, 100 µg/ml spectinomycin). *C. autoethanogenum* DSM 10061 was purchased from the Deutsche Sammlung von Mikroorganismen und Zellkulturen GmbH, Braunschweig, Germany, and cultivated under strict anaerobic conditions in CaGM medium.

CaGM growth medium contains (per liter) 0.25 g of NH_4_Cl, 0.1 g of KCl, 0.2 g of KH_2_PO_4_, 0.2 g of MgSO_4_ ⋅ 7H_2_O, 0.02 g of CaCl_2_ ⋅ 2H_2_O, 1 g of yeast extract, 0.5 ml of 2-g/liter resazurin, 20 g of 2-(*N-*morpholino)ethanesulfonic acid (MES), 0.05 g of Fe(SO_4_)_2_ ⋅ 7H_2_O, 0.25 g of sodium acetate ⋅ 3H_2_O, 0.05 g of nitrilotriacetic acid (NTA), 10 g of fructose (only for heterotrophic growth), 10 ml of a trace element (TSE) solution, and 10 ml of Wolfe’s vitamin solution. The composition of the TSE solution (per liter) was 2 g of nitrilotriacetic acid, 1 g of MnSO_4_ ⋅ H_2_O, 0.8 g of Fe(SO_4_)_2_(NH_4_)_2_ ⋅ 6H_2_O, 0.2 g of CoCl_2_ ⋅ 6H_2_O, 0.2 mg of ZnSO_4_ ⋅ 7H_2_O, 0.02 g of CuCl_2_ ⋅ 2H_2_O, 0.02 g of NaMoO_4_ ⋅ 2H_2_O, 0.02 g of Na_2_SeO_3_, 0.02 g of NiCl_2_ ⋅ 6H_2_O, and 0.02 g of Na_2_WO_4_ ⋅ 2H_2_O. The vitamin solution composition (per liter) was 2 mg of biotin, 2 mg of folic acid, 10 mg of pyridoxine hydrochloride, 5 mg of thiamine HCl, 5 mg of riboflavin, 5 mg of nicotinic acid, 5 mg of calcium pantothenate, 0.1 mg of vitamin B_12_, 5 mg of *p*-aminobenzoic acid, and 5 mg of thioctic acid. The medium was prepared anaerobically, and the pH of the medium was adjusted to 5.8 before sterilization. Prior to inoculation, 100 ml of CaGM medium was reduced with 1 ml of reducing agent 1 (4 g of cysteine HCl/100 ml of water) and 1 ml of reducing agent 2 (7.64 g of NTA, 5.33 g of Na_2_CO_3_, and 8.5 ml of TiCl_3_/100 ml of water).

Cell growth on liquid medium was monitored spectrophotometrically by measuring optical density at 600 nm (OD_600_). Changes in headspace pressure were measured with Rugged Digital Pressure Gauge DPG120 (Omega Engineering). For growth of *C. autoethanogenum* on agar plates, YTF solid medium (10 g/liter fructose, 10 g/liter yeast extract, 16 g/liter tryptone, 0.2 g/liter sodium chloride, 15 g/liter bacteriological agar [Oxoid], pH 5.8), with antibiotics (7.5 µg/ml thiamphenicol, 6 µg/ml clarithromycin) where appropriate, was used. All mutagenesis work was performed inside an anaerobic workstation at 37°C (Don Whitley Scientific Ltd.). For strain comparisons, three or four biological replicates of WT or recombinant *C. autoethanogenum* strains were grown in 250-ml serum bottles containing 50 ml of CaGM medium with 10 g/liter fructose, 200 kPa CO, 10 g/liter fructose plus 200 kPa CO, or 130 kPa H_2_ plus 70 kPa CO_2_ as the growth substrate. Incubation at 37°C was done with agitation (225 rpm) inside New Brunswick Innova shakers (Eppendorf). A standardized 0.5-OD_600_ equivalent of exponentially growing cultures was used as the inoculum.

### DNA manipulation.

DNA manipulation and cloning were carried out according to standard techniques described by Sambrook and Russell (42). Genomic DNA from *C. autoethanogenum* was isolated with a DNeasy Blood and Tissue kit (Qiagen) for PCR diagnostics. For Southern blot analysis, genomic DNA of *C. autoethanogenum* was extracted as described by Bertram and Dürre (43). Plasmid DNA from *C. autoethanogenum* was isolated with a QIAprep Spin Miniprep kit (Qiagen) with the supplementation of 20 mg/ml chicken lysozyme into lysis buffer and incubation at 37°C for 30 min before proceeding to downstream procedures. PCR was carried out with Phusion DNA polymerase (NEB) or Q5 DNA polymerase (NEB). For the primers used in this study, see Table S3 in the supplemental material. Primers were designed with Geneious (Biomatters) and synthesized by Sigma-Aldrich or Eurofins. Sanger sequencing of plasmids and amplicons was carried out by Source Bioscience (United Kingdom).

### Plasmid vectors.

All of the plasmids used in this study were derived from the pMTL80000 series of modular *E. coli-Clostridium* shuttle vectors (44) (see Table S4 in the supplemental material). For the construction of plasmid pMTL83151-P_acsA_, the promoter region of *C. autoethanogenum* acsA (CAETHG_1621) was amplified with oligonucleotides P_acsA_-NotI-F and P_acsA-_NdeI-R and then cloned into plasmid pMTL83151 (44) by using the NotI and NdeI restriction sites. To construct the *acsA* overexpression/complementation plasmid, *acsA*^full^ (CAETHG1620-1621) was first subjected to SOE-PCR (45) with oligonucleotides listed in Table S3 in the supplemental material to remove an internal NdeI site at nucleotide position 342 of CAETHG_1620, resulting in a change in the nucleotide sequence from CATATG to CACATG while retaining the same encoded amino acid sequence. Following cleavage with NdeI and SacI, this amplicon was cloned into plasmid pMTL83151-P_acsA_ to generate plasmid pMTL83151-P_acsA_-acsA^full^.

A FLAG tag sequence (encoding the amino acid sequence DYKDDDDK) was fused to either the N or the C terminus of *acsA*, which was then cloned into plasmid pMTL83151 to generate four plasmid variants to examine the *C. autoethanogenum* acsA translation pattern. The first plasmid, pMTL83151-P_acsA_-FLAG-acsA(TGA), has an N-terminally FLAG-tagged *acsA* gene. It was constructed by initial PCR amplification of native *acsA* with oligonucleotides NcoI-FLAG-acsA-F and acsA-HindIII-R and cloning of the fragment generated into plasmid pMTL83151 by using the NcoI and HindIII restriction sites. This was followed by PCR amplification of a DNA fragment encompassing the native P_acsA_ promoter with primers P_acsA_-SacI-F and P_acsA_-NcoI-R and its cloning between the SacI and NcoI sites of plasmid pMTL83151. The second plasmid, pMTL83151-P_acsA_-acsA(TGA)-FLAG, was generated by the amplification of *acsA* and its native promoter with oligonucleotides P_acsA_-SacI-F and acsA-FLAG-BamHI-R and cloning of the product into plasmid pMTL83151 by using the SacI and BamHI restriction sites.

The third plasmid, pMTL83151-P_acsA_-acsA(TCA)-FLAG, has the internal TGA stop codon of *C. autoethanogenum* acsA mutated to a TCA serine codon. To assemble this plasmid, SOE-PCR was performed with oligonucleotides P_acsA_-SacI-F, acsA(TCA)-SOE-B, acsA(TCA)-SOE-C, and acsA-FLAG-BamHI-R and then the product was cloned into plasmid pMTL83151 by using the SacI and BamHI restriction sites. Similarly, the fourth plasmid, pMTL83151-P_acsA_-acsA(TAA)-FLAG, consists of an *acsA* variant that has the internal TGA codon mutated to another stop codon, TAA. This plasmid was constructed by first performing SOE-PCR with primers P_acsA_-SacI-F, acsA(TAA)-SOE-B, acsA(TAA)-SOE-C, and acsA-FLAG-BamHI-R and then cloning the product into plasmid pMTL83151 by using the SacI and BamHI restriction sites. The cloned promoter and CDS insert in all of the above-described plasmids were verified by Sanger sequencing.

For the construction of ClosTron retargeting plasmids, the appropriate intron-targeting regions within *cooS1*, *cooS2*, and *acsA* were generated *in silico* as previously described (18) using a web-based Perutka algorithm (46). DNA 2.0, Inc., then synthesized the 344-bp intron-targeting region and cloned it into ClosTron vector pMTL007C-E2 (18) by using restriction sites HindIII and BsrGI, resulting in plasmids pMTL007C-E2::cooS1_601s, pMTL007C-E2::cooS2_529s, and pMTL007C-E2::acsA_143s (see Table S4 in the supplemental material).

### Plasmid transfer into *C. autoethanogenum.*

Plasmids were transformed into *E. coli* donor strain CA434 and then conjugated into *C. autoethanogenum* by previously established methods (15, 47, 48). Thiamphenicol (7.5 µg/ml) was used to select for *catP*-based plasmids. Trimethoprim (10 µg/ml) was used to counterselect against *E. coli* CA434 after conjugation. For the validation of plasmid overexpression and plasmid complementation strains, plasmids were isolated from *C. autoethanogenum* transconjugants and subsequently transformed into *E. coli* cells before restriction digestion analysis of the rescued plasmids was carried out (see Fig. S6 in supplemental material). The 16S rRNA gene was also amplified from the genomic DNA of transconjugants with oligonucleotides univ-0027-F and univ-1492-R and then Sanger sequenced for verification.

### Construction of *C. autoethanogenum* ClosTron strains.

Following conjugation of ClosTron retargeting plasmids into *C. autoethanogenum* by using *E. coli* strain CA434 as the donor, thiamphenicol- and trimethoprim-resistant colonies were transferred onto solid YTF medium supplemented with 6 µg/ml clarithromycin to select for intron insertions at target loci and repeatedly streaked onto the same selective medium until plasmid loss was demonstrated, as evident in loss of the ability to grow on medium supplemented with thiamphenicol. Genomic DNA was extracted from the clarithromycin-resistant colonies and subjected to PCR screening with locus-specific flanking primers (see Table S3 in the supplemental material) to identify clones that produced an amplicon 1.8 kb larger than that of the WT control (indicative of ClosTron insertion at specified DNA locus). Sanger sequencing of the ClosTron amplicons was performed to validate the location of ClosTron insertion. As final verification, Southern blot analysis was performed with a digoxigenin High-Prime DNA labeling and detection kit (Roche) as instructed by the manufacturer to ensure that only one ClosTron insertion had occurred in each KO strain. Clones with multiple ClosTron insertions were omitted from downstream studies. For the complementation of the *acsA* KO strain, plasmid pMTL83151-P_acsA_-acsA^full^ was conjugated into this strain and verified by restriction digestion analysis of rescued plasmids from the transconjugants (data not shown).

### Preparation of crude lysates.

Transformed *E. coli* strain XL1-Blue MRFʹ and *C. autoethanogenum* were cultured in 50-ml Falcon tubes inside an anaerobic cabinet. Transformed *E. coli* was cultivated in 50 ml of LB medium supplemented with 20 mM glucose, 10 µM Na_2_SeO_3_, and 25 µg/ml chloramphenicol for 23 to 28 h. *C. autoethanogenum* transconjugants were cultivated in 10 ml of YTF medium supplemented with 15 µg/ml thiamphenicol for 44 to 51 h.

Cell pellets were harvested by centrifugation at 4°C at 7,197 × *g* for 10 min and then resuspended in 300 µl of lysis buffer (50 mM Tris-HCl [pH 7.4], 100 mM NaCl) containing fresh 20 mg/ml chicken lysozyme. Following incubation at 37°C for 45 min, the cell suspensions were sonicated with a Bioruptor Plus (Diagenode) for 60 cycles of 30 s of sonication and 30 s of rest per cycle at 4°C. Following ultracentrifugation at 20,238 × *g* for 5 min at 4°C, the supernatant was harvested as the soluble fraction, whereas the pellet represented the insoluble fraction and was resuspended in 300 µl of lysis buffer without chicken lysozyme. Protein contents were quantified with Bradford reagent (Sigma-Aldrich) with bovine serum albumin as the standard. Both the soluble and insoluble fractions of crude lysates were stored at −20°C for further analysis.

### Western blot analysis.

Cell lysates and purified proteins were analyzed by sodium dodecyl sulfate-polyacrylamide gel electrophoresis (SDS-PAGE). NuPAGE LDS sample buffer (Invitrogen) and 83.3 mM (final concentration) dithiothreitol were added to each sample, and it was boiled at 100°C for 5 min to denature the proteins. The samples and Precision Plus Protein Kaleidoscope Standards (Bio-Rad Laboratories) were then loaded onto 4 to 12% NuPAGE Bis-Tris gels (Invitrogen) in the XCell SureLock Mini-Cell Electrophoresis System (Life Technologies), and NuPAGE MES running buffer (Invitrogen) was added to cover the electrodes. The samples were then subjected to electrophoresis at 150 V for 140 min to separate the proteins, after which the gel was removed from the cast, laid onto Amersham Hybond ECL nitrocellulose membrane (GE Healthcare), and then fitted into an XCell II Blot Module (Life Technologies). To transfer proteins to the membrane, Novex transfer buffer (10% [vol/vol] methanol) was added to the blot module and subjected to electrophoresis at 30 V for 2 h.

Following disassembly of the blot module, the membrane was blocked in 30 ml of TBS buffer (50 mM Tris-HCl, 150 mM NaCl [pH 7.5], 3% [wt/vol] skim milk) at room temperature for 1 h with mild agitation on a shaker. After removal of the used TBS buffer, the membrane (covered in foil to protect it from light) was subjected to overnight incubation in 30 ml of TBS buffer containing 10 µl of anti-FLAG M2-Peroxidase (horseradish peroxidase) monoclonal antibody (Sigma-Aldrich) at room temperature with mild agitation. Following three 5-min washes in TBST (50 mM Tris-HCl, 150 mM NaCl, 0.1% [vol/vol] Tween [pH 7.5]), 4 ml of 3,3′,5,5′-tetramethylbenzidine detection substrate (Sigma-Aldrich) was added to the membrane, which was incubated at room temperature for 5 min before gentle rinsing with deionized H_2_O. The membrane was air dried for 30 min before an image was captured with an EOS 600D DSLR camera (Canon).

### Analytical chemistry.

Analysis of metabolites was performed with the Varian ProStar HPLC (high-performance liquid chromatography) system equipped with a refractive index detector operated at 30°C and an Aminex HPX-87H column (1,300 by 7.8 mm; particle size, 9 µm; Bio-Rad Laboratories) kept at 30°C. Slightly acidified water was used (0.005 M H_2_SO_4_) as the mobile phase with a flow rate of 0.5 ml/min. To remove proteins and other cell residues, samples were centrifuged at 20,238 × *g* for 5 min and the supernatant was filtered with Spartan 13/0.2 RC filters. Ten microliters of the supernatant was then injected into the HPLC system for analysis. Measurements of headspace gas composition were carried out on a Varian CP-4900 micro gas chromatograph with two installed channels. Channel 1 was a 10-m Mol-sieve column running at 70°C with 200 kPa argon and a backflush time of 4.2 s, while channel 2 was a 10-m PPQ column running at 90°C with 150 kPa helium and no backflush. The injector temperature for both channels was 70°C. The run time was set to 120 s, but all of the peaks of interest eluted before 100 s.

### Alignment of *acsA* nucleotide sequences.

The nucleotide sequences of *acsA* from *C. autoethanogenum* (GenBank accession no. NC_022592), *C. ljungdahlii* (CP001666), “*C. ragsdalei*” (HQ876032), *C. carboxidivorans* P7 (HM590563), *C. aceticum* (CP009687), and *C. difficile* 630 (NC_009089) were obtained from NCBI. Multiple global sequence alignments with free-end gaps were performed with Geneious (Biomatters) version 6.1.7.

### Data analysis and presentations.

Statistical analysis was performed and graphically presented results were prepared with GraphPad Prism. Two-tailed, unpaired, parametric Student *t* tests were employed for comparisons of means.

## SUPPLEMENTAL MATERIAL

Figure S1  Screening and validation of *cooS1*, *cooS2*, and *acsA* KO strains. (A) Gel electrophoresis of products of PCRs with exon-spanning primers. Lanes: M, NEB 2-log DNA ladder; 2 to 6, *cooS1* KO clones; 9 to 11, *cooS2* KO clones; 14 to 16, *acsA* KO clones; 1, 8, and 13, nontemplate controls for *cooS1*, *cooS2*, and *acsA* exon-spanning primer pairs, respectively; 7, 12, and 17, WT controls for *cooS1*, *cooS2*, and *acsA* exon-spanning primer pairs, respectively. (B) Southern blot analysis of HindIII-digested genomic DNA of *cooS1* KO clones (lanes 18 to 21), *cooS2* KO clones (lanes 22 to 26), and *acsA* KO clones (lanes 27 to 30). Download Figure S1, TIF file, 1.7 MB

Figure S2  Growth and metabolite profiles of the complemented *acsA* KO strain and plasmid control strains on 200 kPa CO. Panels: A, growth profile; B, acetate profile, C, ethanol profile, D, 2,3-butanediol profile. Black circles, plasmid control (*n =* 4); red squares, *acsA* KO strain (*n =* 3); blue triangles, complemented strain (*n =* 3). Error bars show the standard error of the mean. Download Figure S2, TIF file, 0.2 MB

Figure S3  Effect of *acsA*^full^ overexpression on growth and metabolite profiles of *C. autoethanogenum* on 200 kPa CO. Panels: A, growth profile, B, acetate profile, C, ethanol profile, D, 2,3-butanediol profile. Orange triangles, *acsA*^full^ overexpression strain (*n =* 3); black circles, plasmid control strain (*n =* 4). Error bars show the standard error of the mean. Download Figure S3, TIF file, 0.1 MB

Figure S4  Effect of *acsA*^full^ overexpression on growth and metabolite profiles of *C. autoethanogenum* on 10 g/liter fructose. Panels: A, growth profile, B, acetate profile, C, ethanol profile. Orange triangles, *acsA*^full^ overexpression strain; black circles, plasmid control strain. *n =* 3. Error bars show the standard error of the mean. Download Figure S4, TIF file, 0.1 MB

Figure S5  Nucleotide sequence alignment of *acsA* and internal stop codon in *C. autoethanogenum*. (A) Nucleotide sequence alignment of the *acsA* CDSs (GenBank accession number) of *C. autoethanogenum* (C_auto; NC_022592), *C. ljungdahlii* (C_ljung; CP001666), *C. ragsdalei* (C_rags; HQ876032), *C. carboxidivorans* P7 (C_carb; HM590563), *C. difficile* 630 (C_diff; NC_009089), and *C. aceticum* (C_acetic; CP009687). Variations in the nucleotide sequence of *C. autoethanogenum* are marked in black or as gaps in the gray sequence bar above the red annotations. (B) Inset of panel A highlighting internal the TGA stop codon and putative bacterial selenocysteine insertion sequence (bSECIS) in *acsA* of *C. autoethanogenum*. The image was generated with Geneious 6.1.7 (Biomatters Ltd.). Download Figure S5, TIF file, 1.5 MB

Figure S6  Restriction digestion (SacI and BamHI) analysis of rescued *acsA* plasmids from *C. autoethanogenum* transconjugants. Lanes: M, NEB 2-log DNA ladder; 1 to 4, pMTL83151-P_acsA_-FLAG-acsA(TGA) clones; 5 to 12, pMTL83151-P_acsA_-acsA(TGA)-FLAG clones; 13 to 20, pMTL83151-P_acsA_-FLAG-acsA(TCA) clones; 21 to 24, pMTL83151-P_acsA_-FLAG-acsA(TAA) clones. Download Figure S6, TIF file, 0.9 MB

Table S1  *C. autoethanogenum* CDSs (CAETHG_) with a TGA stop codon that is immediately followed by an in-frame gene. Table S1, DOCX file, 0.02 MB

Table S2  Bacterial strains used in this study. Table S2, DOCX file, 0.02 MB

Table S3  Oligonucleotides used in this study. Table S3, DOCX file, 0.02 MB

Table S4  Plasmids used in this study. Table S4, DOCX file, 0.02 MB
