# Supplementary figures and images for: Insights into CO2 Fixation Pathway of Clostridium autoethanogenum by Targeted Mutagenesis
Source: mBio. 2016 May 24;7(3):e00427-16. doi: 10.1128/mBio.00427-16 (PMC4895105; doi:10.1128/mBio.00427-16)

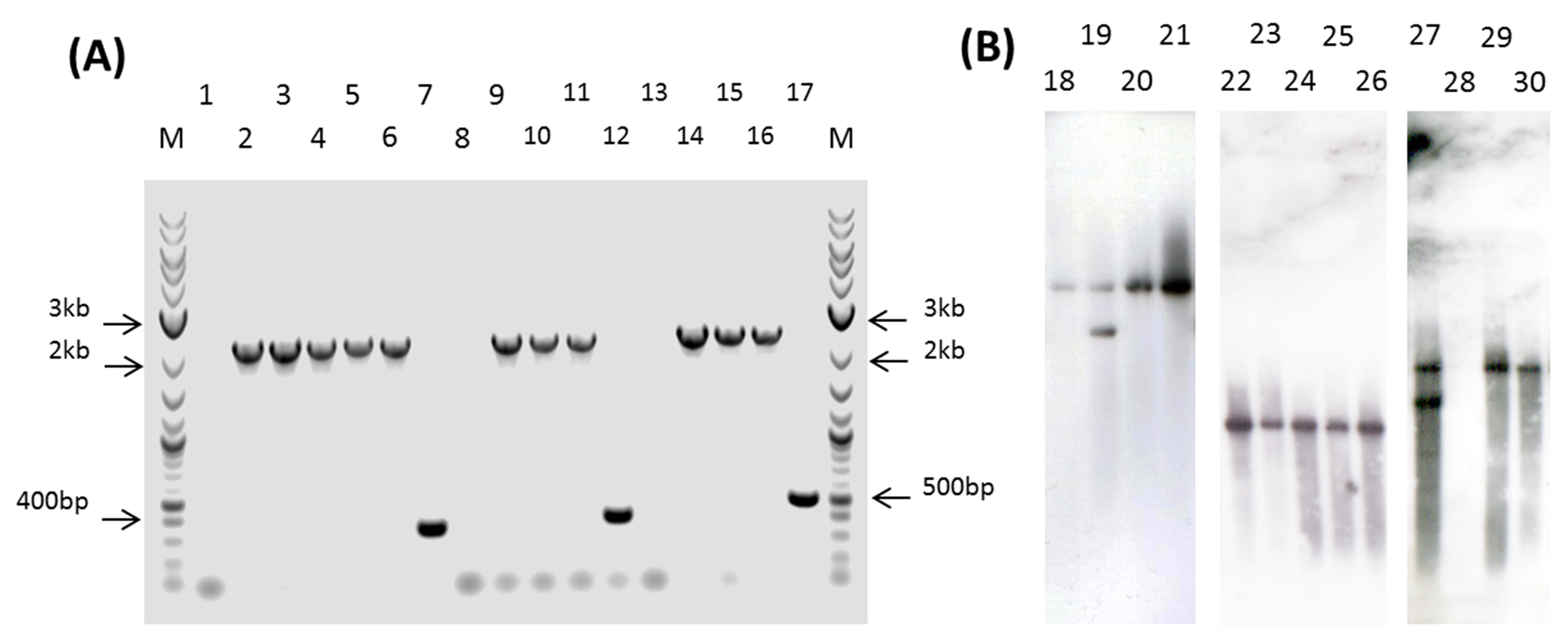

Supplement: Figure S1 — Screening and validation of cooS1, cooS2, and acsA KO strains. (A) Gel electrophoresis of products of PCRs with exon-spanning primers. Lanes: M, NEB 2-log DNA ladder; 2 to 6, cooS1 KO clones; 9 to 11, cooS2 KO clones; 14 to 16, acsA KO clones; 1, 8, and 13, nontemplate controls for cooS1, cooS2, and acsA exon-spanning primer pairs, respectively; 7, 12, and 17, WT controls for cooS1, cooS2, and acsA exon-spanning primer pairs, respectively. (B) Southern blot analysis of HindIII-digested genomic DNA of cooS1 KO clones (lanes 18 to 21), cooS2 KO clones (lanes 22 to 26), and acsA KO clones (lanes 27 to 30). Download [file mbo003162828sf1.tif]

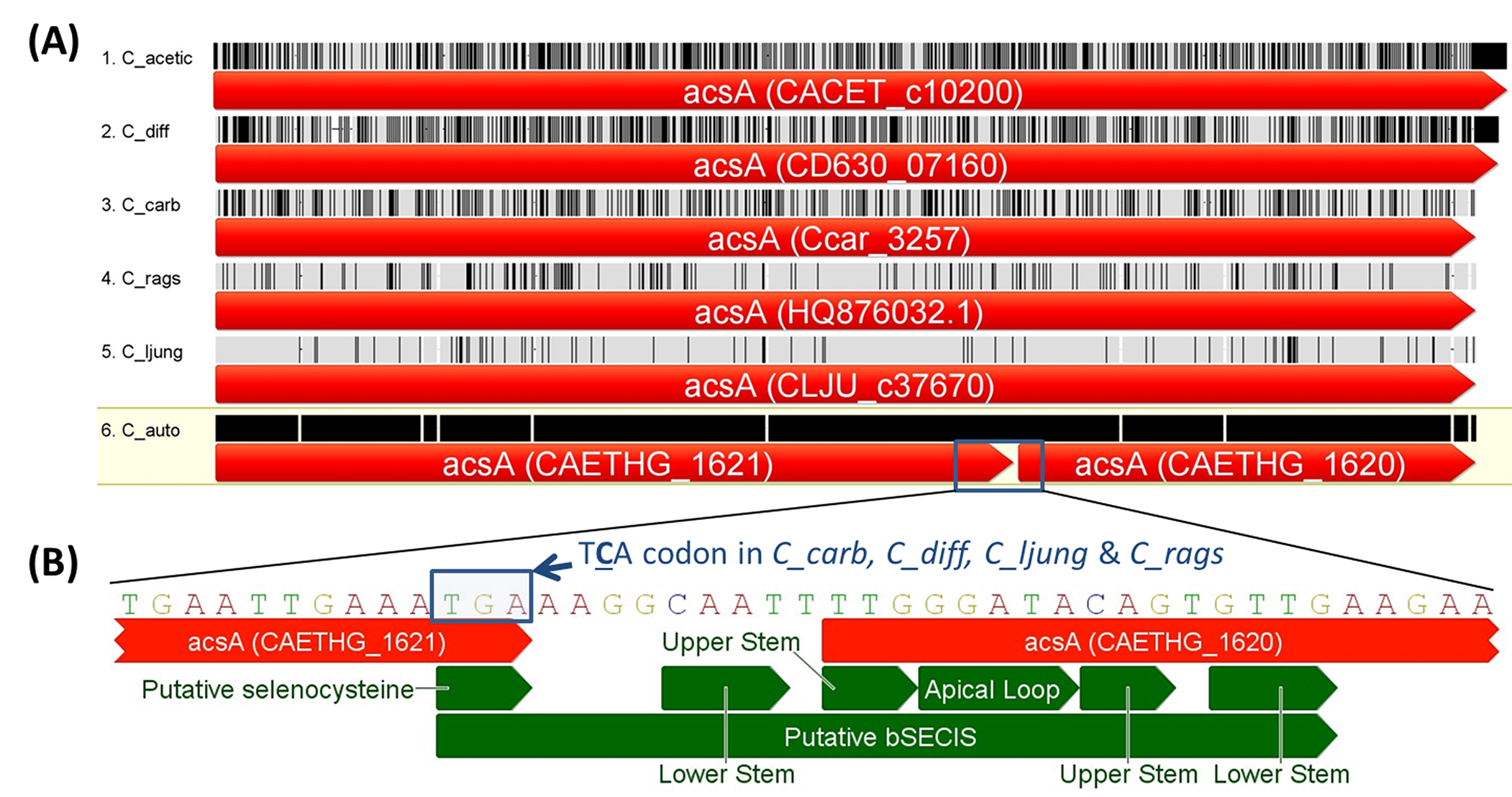

Supplement: Figure S5 — Nucleotide sequence alignment of acsA and internal stop codon in C. autoethanogenum. (A) Nucleotide sequence alignment of the acsA CDSs (GenBank accession number) of C. autoethanogenum (C_auto; NC_022592), C. ljungdahlii (C_ljung; CP001666), C. ragsdalei (C_rags; HQ876032), C. carboxidivorans P7 (C_carb; HM590563), C. difficile 630 (C_diff; NC_009089), and C. aceticum (C_acetic; CP009687). Variations in the nucleotide sequence of C. autoethanogenum are marked in black or as gaps in the gray sequence bar above the red annotations. (B) Inset of panel A highlighting internal the TGA stop codon and putative bacterial selenocysteine insertion sequence (bSECIS) in acsA of C. autoethanogenum. The image was generated with Geneious 6.1.7 (Biomatters Ltd.). Download [file mbo003162828sf5.tif]

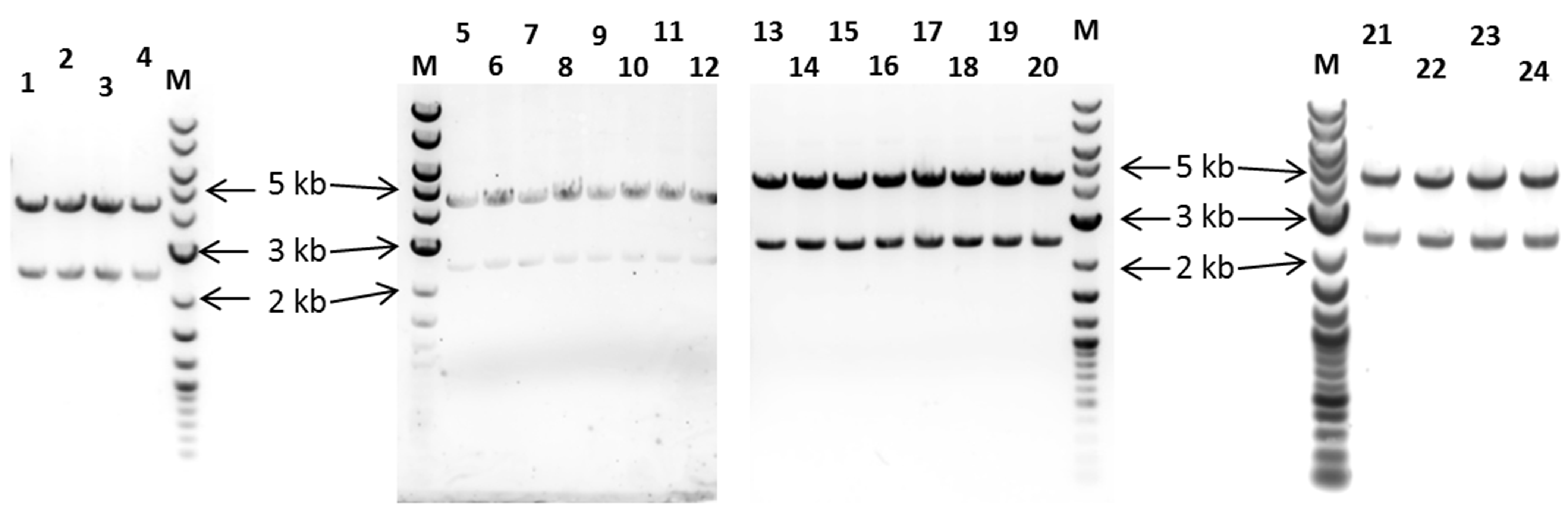

Supplement: Figure S6 — Restriction digestion (SacI and BamHI) analysis of rescued acsA plasmids from C. autoethanogenum transconjugants. Lanes: M, NEB 2-log DNA ladder; 1 to 4, pMTL83151-PacsA-FLAG-acsA(TGA) clones; 5 to 12, pMTL83151-PacsA-acsA(TGA)-FLAG clones; 13 to 20, pMTL83151-PacsA-FLAG-acsA(TCA) clones; 21 to 24, pMTL83151-PacsA-FLAG-acsA(TAA) clones. Download [file mbo003162828sf6.tif]
