# Supplementary material for: Insights into CO2 Fixation Pathway of Clostridium autoethanogenum by Targeted Mutagenesis
Source: mBio. 2016 May 24;7(3):e00427-16. doi: 10.1128/mBio.00427-16 (PMC4895105; doi:10.1128/mBio.00427-16)
Supplement: Table S1 — C. autoethanogenum CDSs (CAETHG_) with a TGA stop codon that is immediately followed by an in-frame gene. [file mbo003162828st1.docx]

Table S1. List of *Clostridium autoethanogenum* CDS (CAETHG_) with ‘TGA’ stop codon which is immediately followed by an in-frame gene.

| CAETHG_ | Gene Annotation | Immediate Downstream Gene Annotation |
| --- | --- | --- |
| 0062 | Integral membrane sensor signal transduction histidine kinase | YbaK/prolyl-tRNA synthetase associated region |
| 0064 | Transcriptional regulator, AraC family | Glyoxalase-like domain containing protein |
| 0085 | Formate Dehydrogenase | Formate Dehydrogenase |
| 0088 | Iron-chelate-transporting ATPase | ABC-type transporter, integral membrane subunit |
| 0149 | Protein of unknown function DUF2284, metal-binding protein | Cobalamin B12-binding domain protein |
| 0397 | CrcB-like protein | CrcB-like protein |
| 0431 | ATP-citrate lyase/succinyl-CoA ligase | Protein of unknown function DUF1116 |
| 0448 | Selenate reductase YgfK | Selenium metabolism protein SsnA |
| 0469 | Hypothetical protein | C_GCAxxG_C_C family protein |
| 0502 | L-aspartate oxidase | Nicotinate-nucleotide pyrophosphorylase |
| 0609 | ABC-type transporter, ATPase component | ABC-type transporter, integral membrane subunit |
| 0615 | TorD-like chaperone | Sulfite reductase, subunit A |
| 0769 | ABC-2 membrane transporter-like protein | GCN5-related N-acetyltransferase |
| 0881 | TadE family protein | Hypothetical protein |
| 1115 | Cobalamin synthesis G domain-containing protein | Cobalt-precorrin-3B C17-methyltransferase |
| 1161 | Transcriptional regulator | Hypothetical protein |
| 1162 | Hypothetical protein | GCN5-related N-acetyltransferase |
| 1174 | Protein of unknown function DUF3786 | Fe-S oxidoreductase |
| 1176 | Phosphoglycerate dehydrogenase | Transcriptional regulator, MarR family |
| 1177 | Drug resistance transporter, EmrB/QacA subfamily | Phosphoglycerate dehydrogenase |
| 1189 | Protein of unknown function DUF4097 | Phospholipid methyltransferase |
| 1403 | Hypothetical protein | Hypothetical protein |
| 1563 | Two component regulator three Y domain-containing protein | Lytic transglycosylase catalytic |
| 1621 | *acsA* | *acsA* |
| 1733 | GerA spore germination protein | Spore germination protein |
| 1861 | MATE efflux family protein | Transcriptional regulator, XRE family |
| 2020 | Hypothetical protein | Radical SAM domain protein |
| 2097 | ATP-dependent protease, Lon family | Replicative DNA helicase |
| 2194 | Hypothetical protein | Hypothetical protein |
| 2510 | Carbamoyl-phosphate synthase large subunit glutamine-dependent | Amidohydrolase |
| 2561 | Transposase IS200-family protein | Hypothetical protein |
| 2582 | Hypothetical protein | ABC transporter related protein |
| 2602 | Glycosyl transferase group 1 | Hypothetical protein |
| 2637 | Mannose-6-phosphate isomerase | dTDP-glucose 4,6-dehydratase |
| 2640 | dTDP-4-dehydrorhamnose reductase | dTDP-4-dehydrorhamnose 3,5-epimerase |
| 2660 | Hypothetical protein | Hypothetical protein |
| 2661 | Hypothetical protein | Von Willebrand factor type A |
| 2734 | Hypothetical protein | GCN5-related N-acetyltransferase |
| 2789 | Formate Dehydrogenase | Formate dehydrogenase |
| 2827 | RNA-binding protein AU-1/Ribonuclease E/G | 50S ribosomal protein L21 |
| 2944 | Hypothetical protein | Spore germination protein |
| 3090 | IMP biosynthesis enzyme PurP domain protein | dTDP-glucose 4,6-dehydratase |
| 3134 | Flagellar hook-basal body protein | Flagellar hook-basal body protein |
| 3181 | Hypothetical protein | Hypothetical protein |
| 3204 | Polyprenyl synthetase | 1-deoxy-D-xylulose-5-phosphate synthase |
| 3237 | Cof-like hydrolase | Metal sensitive transcriptional repressor |
| 3323 | Phosphate ABC transporter, inner membrane subunit PstA | Phosphate ABC transporter, ATPase subunit |
| 3377 | Ribosome biogenesis GTP-binding protein YlqF | Ribonuclease HII |
| 3471 | Electron transfer flavoprotein alpha/beta-subunit | Electron transfer flavoprotein alpha subunit |
| 3635 | Allantoinase | Allantoinase |
| 3749 | UvrD-like DNA helicase | Integrase family protein |
| 3947 | Hypothetical protein | Hypothetical protein |
