# Supplementary material for: Insights into CO2 Fixation Pathway of Clostridium autoethanogenum by Targeted Mutagenesis
Source: mBio. 2016 May 24;7(3):e00427-16. doi: 10.1128/mBio.00427-16 (PMC4895105; doi:10.1128/mBio.00427-16)
Supplement: Table S2 — Bacterial strains used in this study. [file mbo003162828st2.docx]

Table S2. Bacterial strains used in this study.

| Strain | Description/Genotype | Source/Reference |
| --- | --- | --- |
| *Escherichia coli* CA434 | Conjugative transfer strain. Strain HB101 [*thi-I hsdS20* (r_Ƃ_, m_Ƃ_) *supE44 recAB ara-14 leuB5proA2 lacYl galKI* *rpsL20* (Str^R^) *xyl-5 mt1-1*] carrying the Tra^+^, Mob^+^ plasmid R702 [R702-Tc^R^, Sm^R^, Su^R^, Hg^R^] | Williams DR, Young DI, Young M. J. General Microbiology 136:819-826, 1990. |
| *E. coli* XL1-Blue MRF` Kan | Plasmid storage strain. *∆(mcrA)183 ∆(mcrCB-hsdSMR-mrr)173 endA1 supE44 thi-1 recA1 gyrA96 relA1 lac* [*F’proAB lacI^q^Z∆M15* Tn*10* (Tet^R^)] | Stratagene |
| *Clostridium autoethanogenum* DSM 10061 | Wild-type isolate | DSMZ (German Collection of Microorganisms and Cell Cultures GmbH) |
| *C. autoethanogenum*  CauDSM10061-*acsA*143s::CT | Group II intron directionally inserted at *acsA* gene locus (CAETHG_1621; encodes CODH subunit of bi-functional CODH/ACS complex) | This study |
| *C. autoethanogenum* acsAKO+acsA^full^ | Complementation strain of *acsA* KO with plasmid pMTL83151-P_acsA_-acsA^full^ | This study |
| *C. autoethanogenum* CauDSM10061-*cooS1*601s::CT | Group II intron directionally inserted at mono-functional *cooS1* (CAETHG_3005) gene locus | This study |
| *C. autoethanogenum* CauDSM10061-*cooS2*529s::CT | Group II intron directionally inserted at mono-functional *cooS2* (CAETHG_3899) gene locus | This study |
| *C. autoethanogenum* plasmid control strain | *C. autoethanogenum* carrying plasmid pMTL83151-P_acsA_ | This study |
| *C. autoethanogenum* *acsA*^full^ overexpression strain | *C. autoethanogenum* carrying plasmid pMTL83151-P_acsA_-acsA^full^ | This study |
