# Supplementary material for: Insights into CO2 Fixation Pathway of Clostridium autoethanogenum by Targeted Mutagenesis
Source: mBio. 2016 May 24;7(3):e00427-16. doi: 10.1128/mBio.00427-16 (PMC4895105; doi:10.1128/mBio.00427-16)
Supplement: Table S3 — Oligonucleotides used in this study. [file mbo003162828st3.docx]

Table S3. Oligonucleotides used in this study.

| Primer name | DNA sequence (5` to 3`) | Function(s) |
| --- | --- | --- |
| Univ-0027-F | GCG AGA GTT TGA TCC TGG CTC AG | Amplification and sequencing of 16s rRNA for Eubacteria; Weisburg, W. G., Barns, S. M., Pelletier, D. A. & Lane, D. J. *J Bacteriology* 173: 697-703, 1991. |
| Univ-1492-R | CGC GGT TAC CTT GTT ACG ACT T |  |
| P_acsA_-NotI-F | AA**GCGGCCGC**AGATAGTCATAATAGTTCC | Amplification and cloning of *acsA* (CAETHG_1621) promoter region of *C. autoethanogenum* |
| P_acsA-_NdeI-R | TTC**CATATG**AATAATTCCCTCCTTAAAGC |  |
| acsA-143-F | AGGCTACTTTACAATTATTGGACAAGGC | PCR screening for integration of Intron I into *acsA* |
| acsA-143-R | GCCCTTGGATATACACCTAATTTTCTCC |  |
| cooS1-601s-F | TGGAGTGCTGGTGGCCTGTT | PCR screening for integration of Intron I into *cooS1* |
| cooS1-601s-R | AAAAGCTGTACTAGTAGCTGATGCCGT |  |
| cooS2-529s-F | GAGCTGGTACATATAGCCATCATGC | PCR screening for integration of Intron I into *cooS2* |
| cooS2-529s-R | CTGTACCATTTCAAGTCCTATTTGTGC |  |
| acsA-seq-F | GTTATTTCTTTAAGGAGGGAATTATTAAAATGG | Amplification and sequencing of *acsA* (CAETHG_1621 & 1620) |
| acsA-seq-R | AGCTTTTAAGGCATTTTAACTTTGGC |  |
| acsA-seq1 | CCATTGATACAGAGGCAAATCTTGG | Sequencing of *acsA* (CAETHG_1621 & 1620) |
| acsA-NdeI-F | GGGAATTAGC**CATATG**GAAGAAAAAGC | Forward primer for cloning of *acsA* (CAETHG_1621 or CAETHG_1621&1620) |
| acsA-SacI-R | CTTG**GAGCTC**TTCATTTCAATTCAGG | Reverse primer for cloning of part of *acsA* (CAETHG_1621) |
| acsA-SOE-B | CAAGAACCCATGTGAAGTACAGG | SOE PCR to mutate internal NdeI restrition site in downstream CDS of *acsA* (CAETHG_1620) |
| acsA-SOE-C | CCTGTACTTCACATGGGTTCTTG |  |
| acsA-SacI-R2 | ATTT**GAGCTC**TGGCATGGC | Reverse primer for cloning of complete *acsA* (CAETHG_1621 & 1620) |
| P_acsA_-SacI-F | TTCACCA**GAGCTC**GTCATAATAGTTCCAG | Forward primer for amplification and cloning of *C. autoethanogenum acsA* with native promoter |
| acsA(TCA)-SOE-B | CAAAATTGCCTTTGATTTCAATTCAGG | To mutate internal 'TGA' codon of *C. autoethanogenum acsA* into 'TCA' codon |
| acsA(TCA)-SOE-C | TGAATTGAAATCAAAGGCAATTTTGGG |  |
| acsA(TAA)-SOE-B | CAAAATTGCCTTTTATTTCAATTCAGG | To mutate internal 'TGA' codon of *C. autoethanogenum acsA* into 'TAA' STOP codon |
| acsA(TAA)-SOE-C | TGAATTGAAATAAAAGGCAATTTTGGG |  |
| acsA-FLAG-BamH1-R | TT**GGATCC**TTATTATTTGTCATCGTCATCTTTATAATCGATACCTAATTTTTTAC | Reverse primer for amplification and cloning of *C. autoethanogenum acsA* fused with FLAG tag at C-terminus |
| NcoI-FLAG-acsA-F | TTA**CCATGG**ATTATAAAGATGACGATGACAAAGAAGAAAAAGCAAAATC | Amplification and cloning of *C. autoethanogenum acsA* with FLAG tag fused at N-terminus |
| acsA-HindIII-R | TTT**AAGCTT**TGGCATGGCAATTGC |  |
| P_acsA_-NcoI-R | AAT**CCATGG**TAATAATTCCCTCCTTAAAG | Reverse primer for amplification and cloning of native promoter of *C. autoethanogenum acsA* |

Notes: Restriction sites are underlined.
