# Supplementary material for: Insights into CO2 Fixation Pathway of Clostridium autoethanogenum by Targeted Mutagenesis
Source: mBio. 2016 May 24;7(3):e00427-16. doi: 10.1128/mBio.00427-16 (PMC4895105; doi:10.1128/mBio.00427-16)
Supplement: Table S4 — Plasmids used in this study. [file mbo003162828st4.docx]

Table S4. Plasmids used in this study.

| Plasmid | Description | Source/Reference |
| --- | --- | --- |
| pMTL007C-E2 | Clostridial expression vector for the ClosTron, containing a directed Group II intron with *ermB* RAM, flanked by FRT sites, ColE1, pCB102, Cm^R^/Tm^R^ | Heap at al. J Microbiological Methods 70:452-464, 2007. |
| pMTL007C-E2::acsA_143s | ClosTron vector targeting Group II insertional knockout at the *C. autoethanogenum* *acsA* locus (CAETHG_1621) | This work |
| pMTL007C-E2::cooS1_601s | ClosTron vector targeting Group II insertional knockout at the *C. autoethanogenum cooS1* locus (CAETHG_3005) | This work |
| pMTL007C-E2::cooS2_529s | ClosTron vector targeting Group II insertional knockout at the *C. autoethanogenum* *cooS2* locus (CAETHG_3899) | This work |
| pMTL83151 | *E. coli/Clostridium* modular shuttle vector, pCB102, ColE1+tra, Cm^R^/Tm^R^ | Heap et al. J Microbiological Methods 78:79-85, 2009. |
| pMTL83151-P_acsA_ | Overexpression plasmid with *C. autoethanogenum acsA* (CAETHG_1621) promoter cloned between *NotI* and *NdeI* sites | This work |
| pMTL83151-P_acsA_-acsA^full^ | Overexpression plasmid of *C. autoethanogenum acsA* (CAETHG_1621 and CAETHG_1620) | This work |
| pMTL83151-P_acsA_-acsA(TGA)-FLAG | Overexpression plasmid of *C. autoethanogenum acsA* (CAETHG_1621 and CAETHG_1620) with native TGA codon. FLAG tag at C-terminus | This work |
| pMTL83151-P_acsA_-acsA(TCA)-FLAG | Overexpression plasmid of *C. autoethanogenum acsA* (CAETHG_1621 and CAETHG_1620) with mutated TCA codon. FLAG tag at C-terminus | This work |
| pMTL83151-P_acsA_-acsA(TAA)-FLAG | Overexpression plasmid of *C. autoethanogenum acsA* (CAETHG_1621 and CAETHG_1620) with mutated TAA codon. FLAG tag at C-terminus | This work |
| pMTL83151P_acsA_-FLAG-acsA(TGA) | Overexpression plasmid of *C. autoethanogenum acsA* (CAETHG_1621 and CAETHG_1620) with native TGA codon. FLAG tag at N-terminus | This work |
